# Burkholderia humptydooensis sp. nov., a New Species Related to Burkholderia thailandensis and the Fifth Member of the Burkholderia pseudomallei Complex

**DOI:** 10.1128/AEM.02802-16

**Published:** 2017-02-15

**Authors:** Apichai Tuanyok, Mark Mayo, Holger Scholz, Carina M. Hall, Christopher J. Allender, Mirjam Kaestli, Jennifer Ginther, Senanu Spring-Pearson, Molly C. Bollig, Joshua K. Stone, Erik W. Settles, Joseph D. Busch, Lindsay Sidak-Loftis, Jason W. Sahl, Astrid Thomas, Lisa Kreutzer, Enrico Georgi, Jay E. Gee, Richard A. Bowen, Jason T. Ladner, Sean Lovett, Galina Koroleva, Gustavo Palacios, David M. Wagner, Bart J. Currie, Paul Keim

**Affiliations:** a Department of Biological Sciences and The Pathogen and Microbiome Institute, Northern Arizona University, Flagstaff, Arizona, USA; b Menzies School of Health Research, Charles Darwin University, and Northern Territory Medical Program, Royal Darwin Hospital, Darwin, Northern Territory, Australia; c Bundeswehr Institute of Microbiology, Munich, Germany; d Bacterial Special Pathogens Branch, Division of High-Consequence Pathogens and Pathology, Centers for Disease Control and Prevention, Atlanta, Georgia, USA; e Department of Biomedical Sciences, Colorado State University, Fort Collins, Colorado, USA; f Center for Genome Sciences, USAMRIID, Fort Detrick, Maryland, USA; Rutgers, The State University of New Jersey

**Keywords:** Burkholderia humptydooensis sp. nov., Burkholderia pseudomallei complex, MSMB43^T^

## Abstract

During routine screening for Burkholderia pseudomallei from water wells in northern Australia in areas where it is endemic, Gram-negative bacteria (strains MSMB43^T^, MSMB121, and MSMB122) with a similar morphology and biochemical pattern to B. pseudomallei and B. thailandensis were coisolated with B. pseudomallei on Ashdown's selective agar. To determine the exact taxonomic position of these strains and to distinguish them from B. pseudomallei and B. thailandensis, they were subjected to a series of phenotypic and molecular analyses. Biochemical and fatty acid methyl ester analysis was unable to distinguish B. humptydooensis sp. nov. from closely related species. With matrix-assisted laser desorption ionization–time of flight analysis, all isolates grouped together in a cluster separate from other Burkholderia spp. 16S rRNA and *recA* sequence analyses demonstrated phylogenetic placement for B. humptydooensis sp. nov. in a novel clade within the B. pseudomallei group. Multilocus sequence typing (MLST) analysis of the three isolates in comparison with MLST data from 3,340 B. pseudomallei strains and related taxa revealed a new sequence type (ST318). Genome-to-genome distance calculations and the average nucleotide identity of all isolates to both B. thailandensis and B. pseudomallei, based on whole-genome sequences, also confirmed B. humptydooensis sp. nov. as a novel Burkholderia species within the B. pseudomallei complex. Molecular analyses clearly demonstrated that strains MSMB43^T^, MSMB121, and MSMB122 belong to a novel Burkholderia species for which the name Burkholderia humptydooensis sp. nov. is proposed, with the type strain MSMB43^T^ (American Type Culture Collection BAA-2767; Belgian Co-ordinated Collections of Microorganisms LMG 29471; DDBJ accession numbers CP013380 to CP013382).

**IMPORTANCE** Burkholderia pseudomallei is a soil-dwelling bacterium and the causative agent of melioidosis. The genus Burkholderia consists of a diverse group of species, with the closest relatives of B. pseudomallei referred to as the B. pseudomallei complex. A proposed novel species, B. humptydooensis sp. nov., was isolated from a bore water sample from the Northern Territory in Australia. B. humptydooensis sp. nov. is phylogenetically distinct from B. pseudomallei and other members of the B. pseudomallei complex, making it the fifth member of this important group of bacteria.

## INTRODUCTION

Burkholderia species are abundant and occupy diverse ecological niches, including soil, plants, animals, and humans. Probably the most diverse and environmentally adaptable plant-associated bacteria also belong to the genus Burkholderia (1). Many species of Burkholderia have been described since the discovery of B. cepacia by W. H. Burkholder in 1949 as the cause of onion rot (2); this species was later recognized as a human pathogen. Currently, there are more than 90 identified species in this genus (3, 4). There has been a proposal to divide the species into two genera, one of which would retain the Burkholderia name and the other which would be Paraburkholderia gen. nov. (5). At least 20 closely related species belong to the Burkholderia cepacia complex, with many of these soil-dwelling species considered opportunistic pathogens for immunocompromised individuals and other species considered to have both mutualistic and pathogenic roles in plants (6).

Notably, there are two Burkholderia species that can cause severe human and animal diseases: B. pseudomallei and B. mallei, the causative agents of melioidosis and glanders, respectively. B. pseudomallei is a major cause of community-acquired sepsis in northeast Thailand and northern Australia (7). Due to the concerns of their potential use as weapons of mass destruction, federal health agencies in the United States have recently classified these species as Tier 1 (top tier) disease agents (8). It has been well established that B. mallei is a clone of B. pseudomallei that became a host-adapted pathogen in equines, resulting in a massive genome reduction (9). Genetically, both B. pseudomallei and B. mallei are members of the B. pseudomallei phylogenetic group or complex (10). Three additional closely related species have been identified so far in this group: B. thailandensis (11), B. oklahomensis (12), and a newly identified B. thailandensis-like species (13, 14). These closely related species are soil saprophytes and are considered nonpathogenic, although a few strains of B. thailandensis and B. oklahomensis have been described as causing clinical infection in humans (12, 15).

## RESULTS AND DISCUSSION

### Bacterial growth and characteristics.

**FIG 1:**
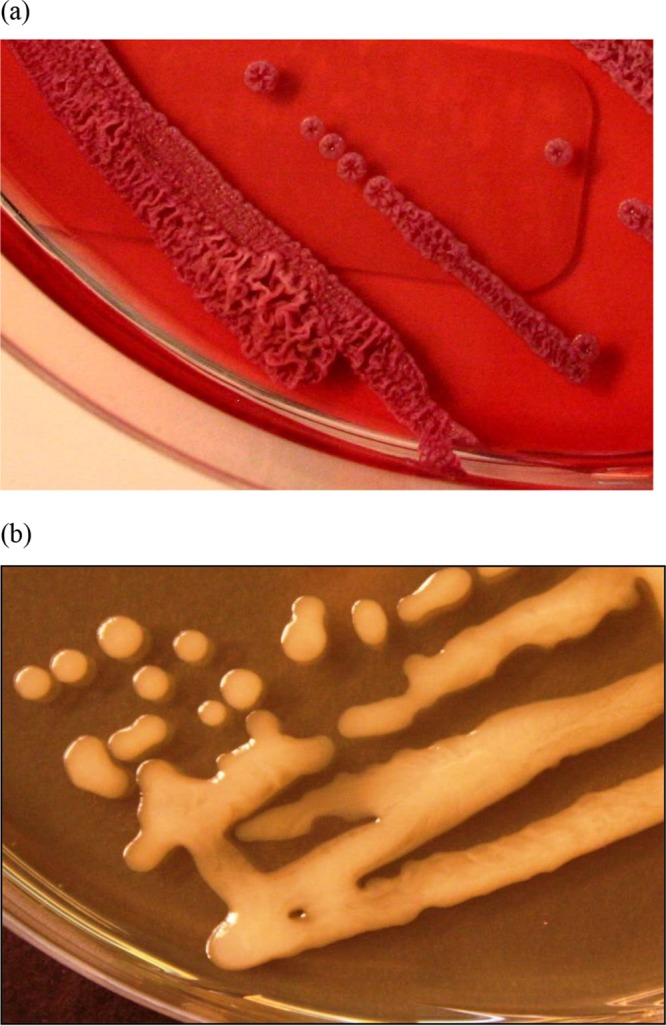
Colony morphology of B. humptydooensis sp. nov. MSMB43^T^. Cultures were grown on Ashdown's agar (a) or on Standard I nutrient agar (b).

As described previously (14), B. humptydooensis sp. nov. MSMB43^T^ did not grow when incubated at temperatures higher than 42°C and also produced little or no gas from nitrate. On Columbia blood agar, smooth and creamy white colonies were observed after 24 h, whereas red, convex, and small (1- to 2-mm) colonies were observed on MacConkey medium after 48 h. Dry and wrinkled colonies were observed on Ashdown's agar after 72 h of growth (Fig. 1), similar to the appearance of B. pseudomallei, while slimy, confluent, honey-like growth appeared on Standard I medium after 48 h (Fig. 1). Variations in colony morphology may exist within MSMB43^T^, as the morphology of MSMB43^T^ on Ashdown's agar was previously reported as smooth and round colonies (16). Bacterial growth was visible on all media after incubation at 25 to 42°C for at least 24 h, with the best growth observed on Columbia blood agar. No growth was observed at 8°C and 45°C. The optimal temperatures for growth were between 28 and 37°C aerobically. All strains showed Gram-negative bipolar staining, appearing as rods of 2 to 3 μm in length and 0.4 to 0.8 μm in diameter. All strains were motile in semisolid media. Biochemical differentiation of B. humptydooensis sp. nov. from B. pseudomallei and B. thailandensis was possible by screening for the presence of tryptophan, esculin, or the assimilation of arabinose (to distinguish from B. pseudomallei) and the assimilation of maltose (to differentiate from B. thailandensis) (Table 1). All strains were positive for nitrate, gelatin, glucose, mannose, mannitol, *N*-acetylglucosamine, gluconate, caprate, adipate, malate, citrate, and phenylacetate. All strains were negative for glucose (acidification) and urea (Table 1).

**TABLE 1:** Phenotypic characteristics of B. humptydooensis sp. nov. and closely related species within the B. pseudomallei group

Biochemical reaction	Characteristic (compound present in medium or assimilated by strain)				
	B. pseudomallei K96243^*a*^	B. thailandensis E264^T^	B. humptydooensis sp. nov. MSMB43^T^	B. humptydooensis sp. nov. MSMB121	B. humptydooensis sp. nov. MSMB122
Tryptophan	+	−	−	−	−
Arginine	+	−	+	−	−
Esculin	−	+	+	+	+
PNPG	−	−	−	−	+
Arabinose assimilation	−	+	+	+	+
Maltose assimilation	−	+	−	−	−

a Data for B. pseudomallei K96243 were obtained from Wuthiekanun et al. (29).

Matrix-assisted laser desorption ionization–time of flight mass spectrometry (MALDI-TOF MS) of the three isolates showed a cluster with other members of the B. pseudomallei complex (see Fig. S1 in the supplemental material). Fatty acid methyl ester analysis was unable to distinguish among the fatty acid profiles from the three B. humptydooensis sp. nov. strains and the closely related species (five B. thailandensis, two B. oklahomensis, and three B. ubonensis strains) (Fig. S2).

### Antimicrobial susceptibility and virulence screening.

Based on the CLSI breakpoints for B. pseudomallei, all strains were determined to be susceptible *in vitro* to ceftazidime, imipenem, trimethoprim-sulfamethoxazole, and doxycycline, whereas resistance to amoxicillin-clavulanic acid was observed (Table 2). The antimicrobial susceptibility pattern of B. humptydooensis sp. nov. generally resembled that of B. pseudomallei (17–20). No significant differences were observed either among the three strains or between the two different susceptibility testing methods. As the maximum concentration of aminoglycosides in the microtiter plates was 32 mg/liter, high-level streptomycin resistance but low-level gentamicin resistance were confirmed using the Etest method (data not shown).

**TABLE 2:** Summary of MICs determined in triplicate by the broth microdilution method

Antimicrobial substance	MIC (mg/liter)		
	MSMB43^T^	MSMB121	MSMB122
Amoxicillin-clavulanic acid^*a*^	32/16	32/16	32/16
Ceftazidime	4	2	4
Imipenem	0.5	0.5	0.5
Rifampin	>8	>8	8
Chloramphenicol	8	4	4
Trimethoprim-sulfamethoxazole	0.5/9.5	≤0.25/4.75	≤0.25/4.75
Streptomycin	>32	>32	>32
Gentamicin	32	32	>32
Doxycycline	1	1	1
Tigecycline	2	4	4
Ciprofloxacin	0.5	1	1
Levofloxacin	0.5	1	1

a Resistance was observed, based upon the CLSI breakpoints of B. pseudomallei.

Neither B. humptydooensis sp. nov. nor B. thailandensis caused mortality in any mice when delivered via the subcutaneous (s.c.) route, nor did any mice show outward signs of illness. In comparison, s.c. infections of fully virulent B. pseudomallei results in 50% mortality within 10 days at a dose of 10^3^ CFU (21). It remains unknown if the inhalation route increases the pathogenicity of species tested in the same way as B. thailandensis E264^T^, which can cause high mortality in mice at doses of 10^4^ to 10^6^ CFU when delivered as an aerosol (22–24).

### Genetic and genomic comparative analyses.

Four rRNA operons are present on the MSMB43^T^ chromosomes, of which two unique versions were found (AQ610_12930/AQ610_01425 and AQ610_21350/AQ610_02540). The genomes of strains MSMB43^T^, MSMB121, and MSMB122 each consisted of two chromosomes (Table 3). These two copies of the 16S rRNA genes were different, which led to ambiguities in conventional sequencing (Fig. S3). The 16S rRNA gene sequence similarities of B. humptydooensis sp. nov. to other members of the B. pseudomallei complex (B. thailandensis, B. mallei, and B. oklahomensis) were 99%. Phylogenetic reconstruction of 16S rRNA and *recA* sequences confirmed genetic proximity to the B. pseudomallei complex but also determined that all B. humptydooensis sp. nov. strains formed their own group within this complex (Fig. S3 and S4).

**TABLE 3:** Whole-genome data^*a*^ for B. pseudomallei group organisms

Species and strain	GC content (%)	Genome size (Mb)	No. of CDS^*c*^
B. humptydooensis sp. nov. MSMB43^T^	67.1	7.3^*b*^	6,324
B. humptydooensis sp. nov. MSMB121	67.5	6.7	5,795
B. humptydooensis sp. nov. MSMB122	67.5	6.8	5,845
B. thailandensis E264^T^	67.6	6.7	5,652
B. oklahomensis C6786^T^	66.9	7.1	6,097
B. pseudomallei K96243	68.1	7.2	5,948
B. mallei ATCC 23344^T^	68.5	5.8	5,006

a Two chromosomes are present in all genomes shown.

b One plasmid present.

c CDS, coding DNA sequences.

All three B. humptydooensis sp. nov. strains, MSMB43^T^, MSMB121, and MSMB122, are sequence type 318 (ST318), and there are no other representatives of this ST. Overall, phylogenetic analysis using multilocus sequence typing (MLST) data supports the separation of B. humptydooensis sp. nov. from the other B. pseudomallei complex members, as described previously (14).

**FIG 2:**
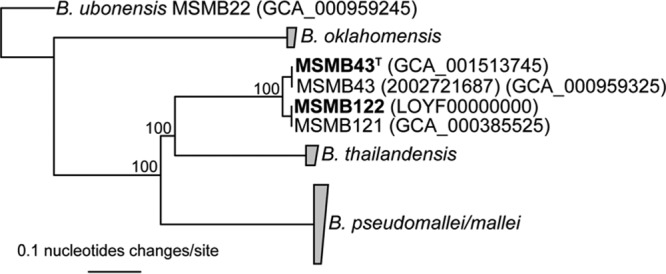
Core genome phylogeny of B. humptydooensis sp. nov. SNPs from the comparison of four B. humptydooensis sp. nov. genomes and representatives of the other closely related species were used to reconstruct the phylogenetic relationships. Genomes from this study are shown in bold and assembly numbers are provided inside parentheses. Numbers at nodes indicate bootstrap support values. Collapsed nodes are shown in gray.

The PacBio sequencing resulted in one finished assembly (for MSMB43^T^) and one mostly finished assembly with 4 contigs (MSMB122) (Table 3). The MSMB43^T^ genome had one circular contig ∼305 kb long that appears to be a plasmid; this same sequence is also present in the previously completed genome of Burkholderia sp. MSMB43^T^ (alternately known as 2002721687 [BioProject no. PRJNA239255]). A comparative genomics approach using large-scale BLAST score ratios (LS-BSR) (25) demonstrated that a large (∼35-kb) stretch of the B. pseudomallei K96243 genome (UniProt gene names BPSS1165 [accession number Q63L43] to BPSS1184 [accession number Q63L24]) on chromosome 2 is highly conserved (>98% identity) in the plasmid sequence, suggesting a shared origin for these regions. The core genome phylogeny demonstrated the position of B. humptydooensis sp. nov. in relation to other clades in the B. pseudomallei complex and confirms the results from other methods (Fig. 2).

Among the three tested B. humptydooensis sp. nov. genomes, the calculated genome-to-genome distance calculation (GGDC) and average nucleotide identity (ANI) values were in the range of 93 to 99% and 98 to 99%, respectively (Table 4). The high GGDC and ANI values indicate that all of these tested strains belong to a single species, including the proposed B. humptydooensis sp. nov. type strain MSMB43^T^. As expected from whole-genome sequencing (WGS) analyses, strain MSMB43^T^ had a slightly lower GGDC similarity (93%) than the other two *B*. humptydooensis sp. nov. strains, which were approximately 97% similar. Further GGDC analysis (Table 4) determined that the similarities of all B. humptydooensis sp. nov. strains to all other tested Burkholderia species in the B. pseudomallei complex were less than 70%, with the highest detected similarity being between B. humptydooensis sp. nov. and B. thailandensis (51.1% [± 3.2%]) (mean ± confidence interval [CI]). This confirmed that the three tested strains are not B. thailandensis but rather a distinct species. The GGDC similarity between B. mallei and B. pseudomallei was 92.5%, which confirmed previous conventional DNA-DNA hybridization (DDH) results and demonstrated that, from a strict taxonomic point of view, they belong to a single species (9).

**TABLE 4:**
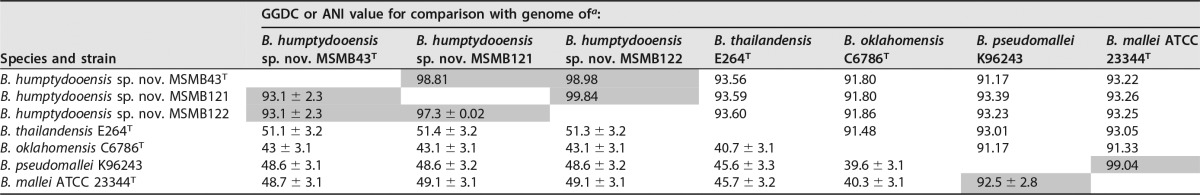
GGDC and ANI values for whole-genome sequence similarities

a Genome-to-genome distance calculations (with confidence intervals) are shown in the bottom left half of the matrix (below the line of identity, i.e., the line formed by blank cells for comparisons of strains with themselves); average nucleotide identities are shown in the top right half of the matrix. Values in shaded boxes represent values above the similarity threshold that defines members of the same species.

In conclusion, we have utilized comprehensive genotyping techniques, including 16S rRNA, *recA*, MLST, and whole-genome-based GGDC, to further support the existence of a new species that is distinct but genetically related to the four members of the B. pseudomallei complex (B. pseudomallei, B. mallei, B. thailandensis, and B. oklahomensis). These analyses confirm the speciation of B. humptydooensis sp. nov., a soil bacterial saprophyte found in the Northern Territory of Australia, where melioidosis is highly endemic. The addition of B. humptydooensis sp. nov. as a new member of the B. pseudomallei complex will benefit evolutionary studies of B. pseudomallei, the serious bacterial pathogen that shares a similar ecological niche with this new species.

## TAXONOMY

Burkholderia humptydooensis sp. nov. (hump.ty.doo.en'sis. L. gen. adj. humptydooensis, pertaining to Humpty Doo, a small town in Northern Territory of Australia, where the first member of this species was isolated).

Bacilli, 0.4 to 0.8 μm in diameter and 2 to 3 μm in length, arranged individually or in irregular clusters. The organism is Gram negative with bipolar staining, motile, and non-spore forming. Growth is observed in a temperature range of 25 to 42°C within 24 to 48 h on various standard solid media. Within 24 h, small colonies (0.5 to 1 mm) are formed on nonselective media (Columbia blood and Standard I) and after 48 h also on selective media (Ashdown's, MacConkey). Best growth occurs at 28 to 37°C after ≥24 h. Colonies become confluent and honey-like in appearance on glycerol-containing medium (Standard I) after 48 h. On Ashdown's selective agar, highly wrinkled purple colonies are observed at ≥48 h, thus resembling the growth of B. pseudomallei.

Assimilation (API 20NE) was found for d-glucose, l-arabinose, d-mannose, d-mannitol, *N*-acetylglucosamine, potassium gluconate, capric acid, adipic acid, malic acid, trisodium citrate, and phenylacetic acid, while results were negative for d-maltose. Esculin and gelatin are hydrolyzed. Variable reactions with l-arginine and 4-nitrophenyl-β d-galactopyranoside (PNPG).

Positive (API ZYM) for alkaline phosphatase, esterase, esterase lipase, lipase, leucine arylamidase, acidic phosphatase, and naphthol-AS-BI-phosphohydrolase. Enzymes absent on API ZYM are valine arylamidase, cystin arylamidase, trypsin, α-chymotrypsin, α- and β-galactosidase, β-glucuronidase, α- and β-glucosidase, *N*-acetyl-β-glucosaminidase, α-mannosidase, and α-frucosidase. This species is aerobic, catalase and oxidase positive, and urease and indole negative. Nitrate and nitrite are reduced (with no gas formation from nitrite) and no production of H_2_S.

B. humptydooensis sp. nov. strains are resistant to aminoglycosides and amoxicillin-clavulanic acid but susceptible to trimethoprim-sulfmethoxazole, doxycycline, imipenem, and ceftazidime. All B. humptydooensis sp. nov. strains are seroreactive with sera from melioidosis patients who were infected with B. pseudomallei serotype B strains. All strains produced O-antigen ladder type B2, except that strain MSMB43^T^ produced a novel O-antigen ladder type (26). The type strain, MSMB43^T^, has been previously referred to as B. thailandensis-like species in multiple studies (13, 14). MSMB43^T^ was isolated in 1995 from an automated water well (bore) in Humpty Doo, Australia. B. humptydooensis sp. nov., like B. thailandensis, is nonvirulent in mice. In addition, MSMB43^T^ is known to produce thailanstatins, which possess antiproliferative activities in representative human cancer cell lines (27). The type strain MSMB43^T^ has been deposited in the American Type Culture Collection as BAA-2767 and the Belgian Co-ordinated Collections of Microorganisms as LMG 29471.

## MATERIALS AND METHODS

### Strain isolation.

Strain MSMB43^T^ was isolated from a water sample from an automated water bore (well) collected in 1995 and examined for B. pseudomallei in the Northern Territory (NT) of Australia. This strain was initially thought to be B. thailandensis due to its ability to assimilate arabinose as a sole carbon source, which is a trait used to discriminate B. thailandensis from B. pseudomallei (arabinose negative) (14). The bore from which MSMB43^T^ was discovered is located in Humpty Doo, a region of rural properties outside the capital of the NT, Darwin. The Top End of the NT has a high incidence rate of melioidosis (28). In fact, the water sample from which MSMB43^T^ was recovered also yielded B. pseudomallei. An additional two strains (MSMB121 and MSMB122) were both isolated in 2007 from a single separate bore water sample within the NT collected approximately 950 km south of the territory capital, Darwin, resulting in a 910-km separation between the two sample sites of MSMB121/MSMB122 and MSMB43^T^. To date, the proposed B. humptydooensis sp. nov. has not been identified outside the NT, and it has not been isolated from any clinical specimens from patients within the NT.

The specific epithet “humptydooensis” given to this new species was adopted from the location name Humpty Doo, where this new species was first discovered.

### Bacterial growth and characteristics.

All three strains were grown at temperatures of 8, 25, 37, 42, and 45°C for 24, 48, 72, and 144 h on Columbia blood agar, MacConkey agar, Ashdown's selective agar, and Standard I nutrient agar with and without supplementary CO_2_. Cell morphology was examined using a Zeiss light microscope at 1,000× magnification with cells grown for 2 days at 37°C. Biochemical data were obtained for all three strains of B. humptydooensis sp. nov. (MSMB43^T^, MSMB121, and MSMB122) and compared to data for strains of B. pseudomallei (K96243) (29) and B. thailandensis (E264^T^) by using the API ONE and API Zym systems (bioMérieux) according to the manufacturer's instructions.

MALDI-TOF MS and fatty acid methyl ester analysis was performed for all three B. humptydooensis sp. nov strains (see the text in the supplemental material for a detailed description of these methods).

### Antimicrobial susceptibility screening.

MICs were determined by the broth microdilution method using commercially available CE-certified Micronaut-S 96-well microtiter plates (Merlin, Bornheim-Hersel, Germany) containing 2-fold serial dilutions of the following antibiotics: amoxicillin-clavulanic acid (0.5 to 64/0.25 to 32 mg/liter), ceftazidime (0.5 to 64 mg/liter), imipenem (0.25 to 32 mg/liter), rifampin (0.0625 to 8 mg/liter), chloramphenicol (0.5 to 64 mg/liter), trimethoprim-sulfamethoxazole (0.25 to 32/4.75 to 608 mg/liter), streptomycin (0.25 to 32 mg/liter), gentamicin (0.25 to 32 mg/liter), doxycycline (0.25 to 32 mg/liter), tigecycline (0.03125 to 4 mg/liter), ciprofloxacin (0.03125 to 4 mg/liter), and levofloxacin (0.0625 to 4 mg/liter). One well without antibiotic was used as a growth control. All plates containing the lyophilized antimicrobial substances were stored at room temperature until use.

Testing conditions were in accordance with the current Clinical and Laboratory Standards Institute (CLSI) recommendations for B. pseudomallei (30). Single colonies of MSMB43^T^, MSMB121, and MSMB122 were picked from agar plates and inoculated in physiological saline (0.85% NaCl) until the turbidity matched that of a 0.5 McFarland standard. The suspension was diluted 221-fold in cation-adjusted Mueller-Hinton II broth (catalog number 297701; Becton Dickinson). After incubation for 24 h at 37°C in a 5% CO_2_ atmosphere, bacterial growth was verified photometrically at a wavelength of 620 nm using a commercial photometer (Merlin, Bornheim-Hersel, Germany), and each strain was tested in triplicate. Additionally, a gradient strip method (Etest; bioMérieux) was applied to investigate a broader range of antibiotic concentrations.

### Virulence testing in mouse models.

The pathogenic potential of B. humptydooensis sp. nov. MSMB43^T^ was investigated in a BALB/c mouse model and compared to the pathogenic potential of B. thailandensis (type strain E264^T^). Live cultures were grown to logarithmic phase (optical density at 600 nm, ∼1.0) in Luria-Bertani (LB) broth as previously described (22). Sterile 1× phosphate-buffered saline (PBS) was used to wash cells twice before making dilutions for injecting mice. Viability counts of the final inocula were made on LB agar plates. Six- to 8-week-old female BALB/c mice in treatment groups of 5 mice per cage were used. Food and water were provided *ad libitum*. All mice in a single cage received the same infectious dose (B. humptydooensis sp. nov.: 1.05 × 10^4^, 10^5^, or 10^6^ CFU; B. thailandensis: 3.4 × 10^4^, 10^5^, or 10^6^ CFU) via a single s.c. injection in the scruff of the neck. Mice were monitored daily for health status. All mice were euthanized on day 21 postinjection. This work was conducted under approved protocols from the NAU IACUC (protocol 14-011) and DOD ACURO (HDTRA1-12-C-0066_Wagner).

### 16S rRNA and *recA* gene analysis.

16S rRNA and *recA* gene sequencing analyses were performed on three B. humptydooensis sp. nov. strains: MSMB43^T^, MSMB121, and MSMB122, as previously described (11, 31). From whole-genome analysis of strain MSMB43^T^ using the SSU-ALIGN program (32), we investigated the number of rRNA operons present. Phylogenetic reconstruction of 16S rRNA and *recA* sequences was conducted using MEGA version 6 (33).

### MLST.

MLST was performed on all three B. humptydooensis sp. nov. strains as previously described (9). As of 7 October 2016, a total of 1,439 sequence types had been identified in B. pseudomallei and closely related species by MLST (http://www.MLST.net). The seven genes that comprise this MLST are *ace*, *gltB*, *gmhD*, *lepA*, *lipA*, *narK*, and *ndh*.

### Genome assembly and core genome phylogeny.

Two genomes (MSMB43^T^ and MSMB122) were sequenced on the PacBio platform. Two other genomes that group with B. humptydooensis sp. nov. are present in GenBank and consist of strains MSMB121 and MSMB43^T^ (which is the same type strain used in this study but is listed with an alternative BioProject strain identifier, 2002721687 [BioProject no. PRJNA239255]) (34, 35) with GenBank assembly accession numbers GCA_000385525 and GCA_000959325, respectively. A comparative genomics approach using LS-BSR (25) was also performed with the B. pseudomallei genome strain K96243 (BPSS1165 to BPSS1184).

For the core genome phylogeny, genomes were aligned against B. pseudomallei K96243 by using NUCmer (36). The reference genome was also aligned against itself to identify duplicated regions, which were masked from subsequent analyses; the NASP pipeline was used to wrap these methods (http://tgennorth.github.io/NASP/). A phylogeny was inferred by using RAxML v8 (37) on a large set (*n* = 331,000) of concatenated single nucleotide polymorphisms (SNPs) using a time-reversible model incorporating the Lewis ascertainment bias correction.

### Genome-to-genome distance calculations.

DDH is the current gold standard for bacterial species delineation. DDH is necessary for the description of a new species within a taxon when strains share more than 98.65% 16S rRNA gene sequence similarity (38). If DNA-DNA relatedness between two strains is less than 70%, the two strains are considered different species. However, DDH is laborious and difficult to standardize, and interlaboratory reproducibility is relatively low. DDH was previously performed on MSMB43^T^ and showed a relative binding ratio of 91% with B. thailandensis (ATCC 700388) with a divergence of 4% (14). Because of the drawbacks of conventional DDH and the rapid progress in genome sequencing techniques, various *in silico* algorithms for calculating genome-to-genome similarities or distances have been developed. In addition to the commonly used ANI method (39), recently a highly reliable estimator for the relatedness of genomes was developed by Jan P. Meier-Kolthoff and colleagues (40). GGDC produces digital DDH values that correlate well with values obtained by conventional DDH, which is of utmost importance for compatibility with the current species concept and also provides confidence interval estimation. DDH values were calculated using formula 2 in GGDC; this formula summed the identities found in high-scoring segment pairs (HSP) and then divided them by the overall HSP length. The GGDC service is available from the German Collection of Microorganisms and Cell Cultures home page (http://ggdc.dsmz.de/distcalc2.php). PacBio assemblies were used to determine the distances among B. humptydooensis sp. nov. and other closely related species. The genomes were subjected to GGDC analysis and compared to the available genome sequences of B. pseudomallei, B. mallei, B. oklahomensis, and B. thailandensis reference strains (K96243, ATCC 23344^T^, C6786^T^, and E264^T^, respectively). For comparison, the ANI values were also calculated for all reference sequences by using JSpecies (41); the authors of JSpecies determined that ANI values of <95% indicate separate species.

### Accession number(s).

GenBank accession numbers for the 16S rRNA gene sequences of B. humptydooensis sp. nov. strains MSMB121 and MSMB122 are KF378608 and KF378609, respectively. The complete whole-genome sequence of the strain MSMB121 was published under GenBank accession numbers CP004095 and CP004096 (34, 35). The assembly for MSMB43^T^ was published under GenBank assembly number GCA_001513745 and that for MSMB122 under SRA numbers SRR1956040 and LNPD00000000.

## Supplementary Material

Supplemental material
