# Supplementary material for: Burkholderia humptydooensis sp. nov., a New Species Related to Burkholderia thailandensis and the Fifth Member of the Burkholderia pseudomallei Complex
Source: Appl Environ Microbiol. 2017 Feb 15;83(5):e02802-16. doi: 10.1128/AEM.02802-16 (PMC5311406; doi:10.1128/AEM.02802-16)
Supplement: Supplemental material [file supp_83_5_e02802-16__index.html]

Supplemental material 

# Burkholderia humptydooensis sp. nov., a New Species Related to Burkholderia thailandensis and the Fifth Member of the Burkholderia pseudomallei Complex

## Supplemental material

- Supplemental file 1 -

  Detailed methods: MALDI-TOF and FAME profile analysis; dendrogram demonstrating strain relatedness revealed by MALDI-TOF analysis (Fig. S1); dendrogram demonstrating the relatedness of fatty acid compositions in *B. humptydooensis* sp. nov. and closely related species (Fig. S2); 16S maximum-likelihood phylogeny using both copies of the 16S rRNA gene from each *B. humptydooensis* sp. nov. strain (Fig. S3); maximum-likelihood phylogeny of the *recA* sequence using 193 sequences (Fig. S4).

  PDF, 452K
