# Supplementary material for: Burkholderia humptydooensis sp. nov., a New Species Related to Burkholderia thailandensis and the Fifth Member of the Burkholderia pseudomallei Complex
Source: Appl Environ Microbiol. 2017 Feb 15;83(5):e02802-16. doi: 10.1128/AEM.02802-16 (PMC5311406; doi:10.1128/AEM.02802-16)
Supplement: Supplemental material [file AEM.02802-16_zam999117680s1.pdf]

MALDI-TOF:

Preparations of bacterial isolates (MSMB43^T, MSMB121, MSMB122) for matrix-assisted laser-desorption/ionization time-of-flight mass spectrometry (MALDI-TOF MS) were done according to the ethanol/formic acid extraction protocol recommended by the manufacturer (Bruker Daltonics) and as previously described (1). Briefly, a loopful of bacterial material was evenly dissolved in 300 µl analytical grade water, and 900 µl pure ethanol was added. The cell suspension was centrifuged at 13,000 × g for 2 minutes, and the supernatant was discarded. The centrifugation was repeated, and the residual ethanol was discarded. The pellet was air dried and thoroughly resuspended in 5-50 µl 70% formic acid depending on the size, and, finally, an equal volume of acetonitrile was added. After centrifugation at 13,000 × g for 2 minutes, 1 µl of the supernatant was transferred to the MALDI target plate. After air-drying at room temperature 1 µl of matrix solution (saturated solution of α-cyano-hydroxy-cinnaminic acid in 50% aqueous acetonitrile containing 2.5% trifluoroacetic acid) per spot were was applied. MALDI-TOF MS was conducted using a Microflex LT mass spectrometer (Bruker Daltonics) equipped with a nitrogen laser. All spectra were recorded in linear, positive ion mode across a mass/charge ratio (m/z) of 2,000 to 20,000. The acceleration voltage was 20 kV. Spectra were collected as a sum of 240 shots across a spot. Main spectra were calculated from 8 spectra per strain and used for construction of a score oriented dendrogram by using the BioTyper software (version 2.3, Bruker Daltonics). In these analyses *B. humptydooensis* sp. nov. strains grouped together with *B. ubonensis* from the Bcc and other members of the *B. pseudomallei* complex (Fig. S1).

Fatty acid methyl ester (FAME) profile analysis:

Fatty acid methyl esters analysis was unable to distinguish among the fatty acid profiles from the three *B. humptydooensis* sp. nov. strains and its closely related species (five *B. thailandensis*, two *B. oklahomensis*, and three *B. ubonensis* strains). The fatty acid methyl esters were performed using the MIDI Sherlock® Microbial Identification System by Microbial ID, Inc. (Delaware, USA). Similarity index analysis was used to display the relatedness of FAME profiles produced by these species. The analysis revealed that FAME profiles of the four *B. humptydooensis* sp. nov. strains grouped into two sub-groups, along with strains from other species (Fig. S2). This finding suggests that *B. humptydooensis* sp. nov. had similar FAME profiles with other tested genetically related *Burkholderia* species and this technique cannot be used to distinguish *B. humptydooensis* sp. nov. or distinguish the other closely related species.

References:

1. **Mellmann A, Cloud J, Maier T, Keckevoet U, Ramminger I, Iwen P, Dunn J, Hall G, Wilson D, Lasala P, Kostrzewa M, Harmsen D.** 2008. Evaluation of matrix-assisted laser desorption ionization-time-of-flight mass spectrometry in comparison to 16S rRNA gene sequencing for species identification of nonfermenting bacteria. J Clin Microbiol **46:**1946-1954.

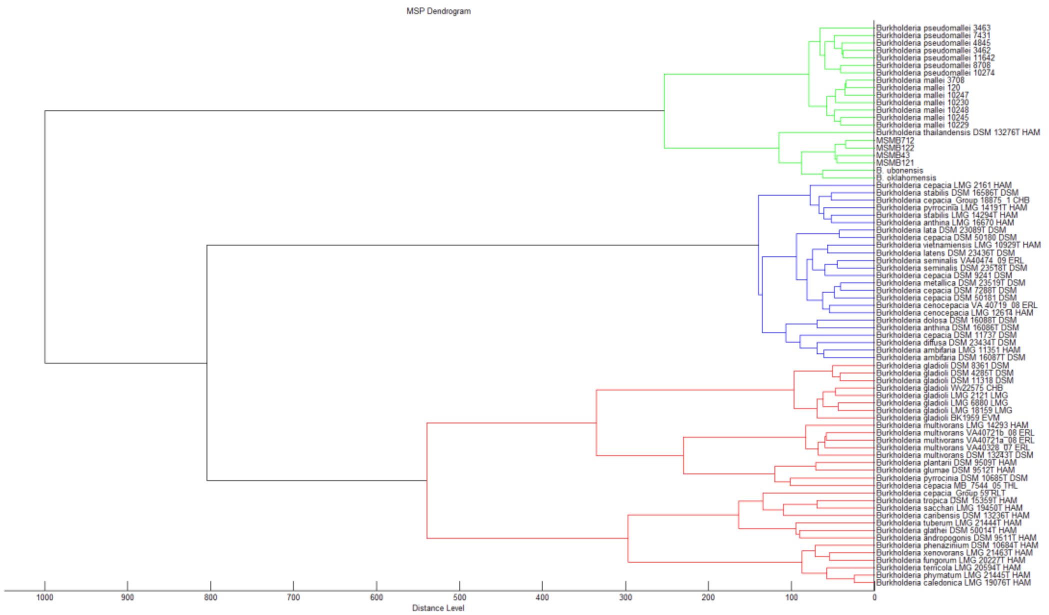

Fig. S1. Dendrogram demonstrating strain relatedness revealed by MALDI – TOF analysis.

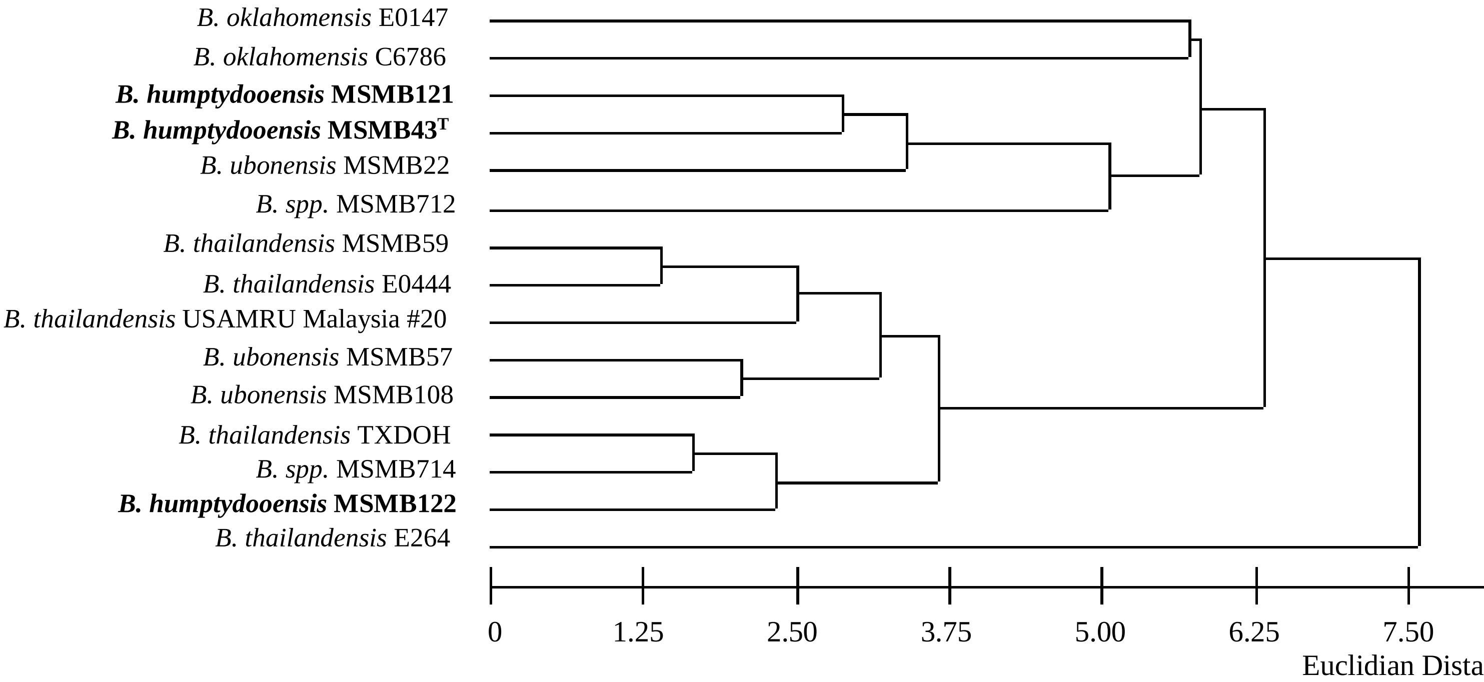

Fig. S2. A dendrogram demonstrating the relatedness of fatty acid compositions in *B. humptydooensis* sp. nov. and other closely related species. Similarity Index in the Microbial Identification System (MIS) was used to display how closely the fatty acid compositions found in *B. humptydooensis* sp. nov., a new species, compared with those from other genetically related species. FAME (Fatty Acid Methyl Esters) profiles were generated using GC-MS. The dendrogram was generated based upon the clustering analysis technique to produce unweighted pair matching based on FAME profiles. Our analysis has shown that FAME profiles from three tested *B. humptydooensis* sp. nov. strains were grouped into two sub-groups. The first group also contained one *B. ubonensis* and two *B. oklahomensis* strains, while the second group contained most of *B. thailandensis* strains and two *B. ubonensis* strains. We noted that *B. thailandensis* E264 had a distinct FAME profile to both subgroups.

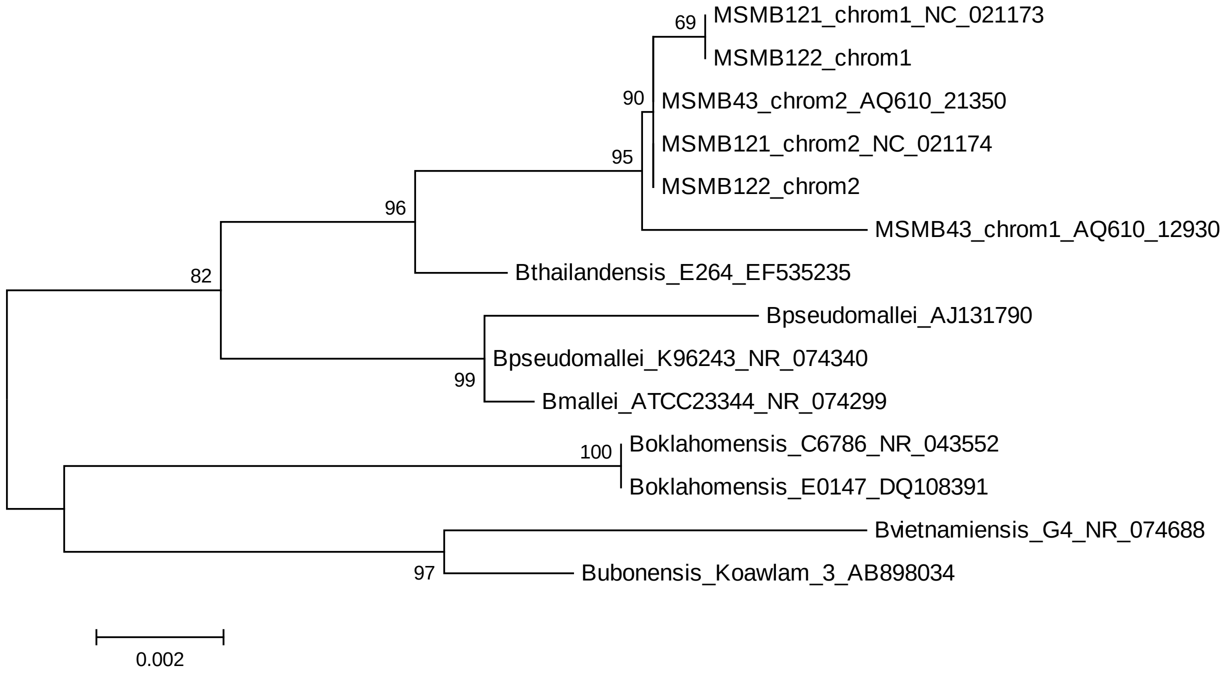

Fig. S3. 16S maximum likelihood (500 bootstrap) phylogeny using both copies of 16S from each *B. humptydooensis* sp. nov. strain (1420 bp and 14 sequences).

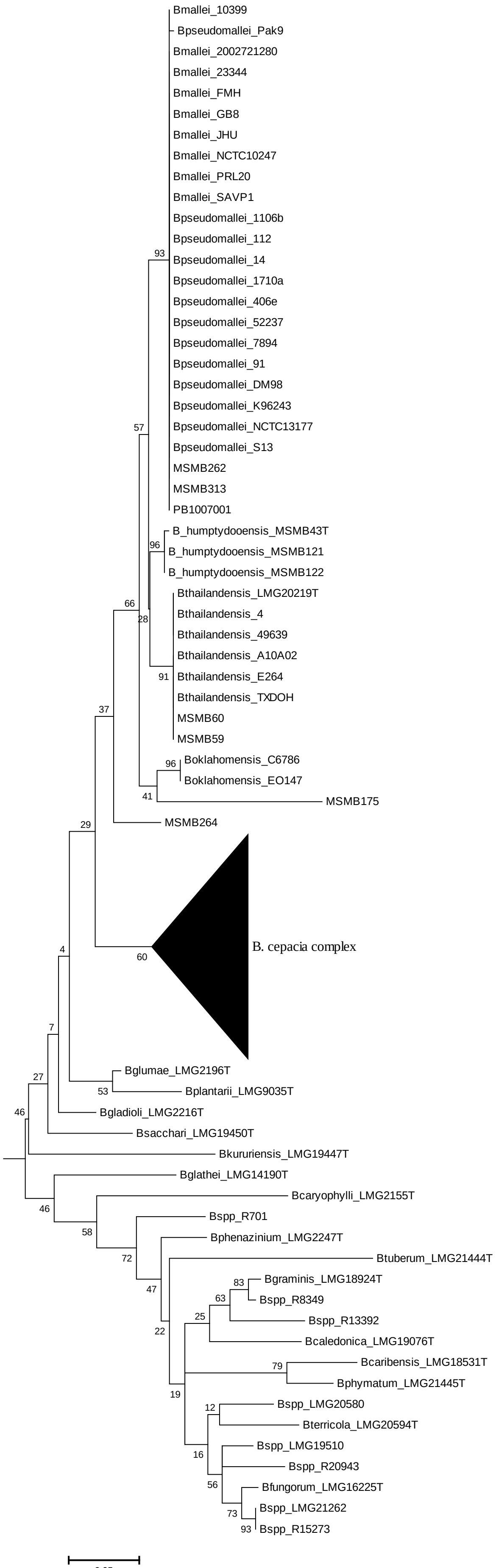
Bmallei_10399
Bpseudomallei_Pak9
Bmallei_2002721280
Bmallei_23344
Bmallei_FMH
Bmallei_GB8
Bmallei_JHU
Bmallei_NCTC10247
Bmallei_PRL20
Bmallei_SAVP1
Bpseudomallei_1106b
Bpseudomallei_112
Bpseudomallei_14
Bpseudomallei_1710a
Bpseudomallei_406e
Bpseudomallei_52237
Bpseudomallei_7894
Bpseudomallei_91
Bpseudomallei_DM98
Bpseudomallei_K96243
Bpseudomallei_NCTC13177
Bpseudomallei_S13
MSMB262
MSMB313
PB1007001
B_humptydooensis_MSMB43T
B_humptydooensis_MSMB121
B_humptydooensis_MSMB122
Bthailandensis_LMG20219T
Bthailandensis_4
Bthailandensis_49639
Bthailandensis_A10A02
Bthailandensis_E264
Bthailandensis_TXDOH
MSMB60
MSMB59
Boklahomensis_C6786
Boklahomensis_EO147
MSMB175
MSMB264
B. cepacia complex
Bglumae_LMG2196T
Bplantarii_LMG9035T
Bgladioli_LMG2216T
Bsacchari_LMG19450T
Bkururiensis_LMG19447T
Bglathei_LMG14190T
Bcaryophylli_LMG2155T
Bspp_R701
Bphenazinium_LMG2247T
Btuberum_LMG21444T
Bgraminis_LMG18924T
Bspp_R8349
Bspp_R13392
Bcaledonica_LMG19076T
Bcaribensis_LMG18531T
Bphymatum_LMG21445T
Bspp_LMG20580
Bterricola_LMG20594T
Bspp_LMG19510
Bspp_R20943
Bfungorum_LMG16225T
Bspp_LMG21262
Bspp_R15273
93
57
96
66
28
91
37
96
41
29
60
4
7
27
46
53
46
58
72
47
22
83
63
25
79
19
12
16
56
73
93
0.05

1

Fig. S4. Maximum likelihood phylogeny (1,500 bootstrap) of recA sequence (335 bp) using 193 sequences.
